# Fluorescent protein vectors for pancreatic islet cell identification in live-cell imaging

**DOI:** 10.1007/s00424-016-1864-z

**Published:** 2016-08-18

**Authors:** Hongyan Shuai, Yunjian Xu, Qian Yu, Erik Gylfe, Anders Tengholm

**Affiliations:** Department of Medical Cell Biology, Uppsala University, Biomedical Centre, Box 571, SE-751 23 Uppsala, Sweden

**Keywords:** Islets, α-cell, β-cell, δ-cell, PP-cell, Insulin, Glucagon, Somatostatin, Pancreatic polypeptide, Ca^2+^, cAMP

## Abstract

The islets of Langerhans contain different types of endocrine cells, which are crucial for glucose homeostasis. β- and α-cells that release insulin and glucagon, respectively, are most abundant, whereas somatostatin-producing δ-cells and particularly pancreatic polypeptide-releasing PP-cells are more scarce. Studies of islet cell function are hampered by difficulties to identify the different cell types, especially in live-cell imaging experiments when immunostaining is unsuitable. The aim of the present study was to create a set of vectors for fluorescent protein expression with cell-type-specific promoters and evaluate their applicability in functional islet imaging. We constructed six adenoviral vectors for expression of red and green fluorescent proteins controlled by the insulin, preproglucagon, somatostatin, or pancreatic polypeptide promoters. After transduction of mouse and human islets or dispersed islet cells, a majority of the fluorescent cells also immunostained for the appropriate hormone. Recordings of the sub-plasma membrane Ca^2+^ and cAMP concentrations with a fluorescent indicator and a protein biosensor, respectively, showed that labeled cells respond to glucose and other modulators of secretion and revealed a striking variability in Ca^2+^ signaling among α-cells. The measurements allowed comparison of the phase relationship of Ca^2+^ oscillations between different types of cells within intact islets. We conclude that the fluorescent protein vectors allow easy identification of specific islet cell types and can be used in live-cell imaging together with organic dyes and genetically encoded biosensors. This approach will facilitate studies of normal islet physiology and help to clarify molecular defects and disturbed cell interactions in diabetic islets.

## Introduction

The pancreatic islets of Langerhans are key regulators of glucose homeostasis. They contain four major types of endocrine cells: α-, β-, δ-, and PP-cells, secreting glucagon, insulin, somatostatin, and pancreatic polypeptide (PP), respectively [36, 45]. The β-cells are most abundant and constitute 60–80 % of the endocrine cells in islets from mice and ~50 % in human islets. α-cells make up ~20 % in mouse and ~40 % in man, while δ- and PP-cells are more scarce constituting <10 and <1 % of the islet cells, respectively. There is also a small fraction of ε-cells secreting ghrelin [54]. Insulin decreases blood glucose levels and insufficient insulin release from the β-cells causes most forms of diabetes mellitus. Glucagon, on the contrary, increases blood glucose. Diabetic patients often show exaggerated glucagon secretion that contributes to hyperglycemia, but also a reduced glucagon response to hypoglycemia, which may become fatal due to the brain’s strong glucose dependence [14, 50]. All major islet hormones are released in a pulsatile manner [15, 21, 24], which promotes hormone sensitivity in the target cells [37, 42]. Indeed, insulin and glucagon pulsatility is typically disturbed in early stages of diabetes [27, 34, 40] but the molecular mechanisms that underlie the dysregulated secretion are unclear. The physiological relevance of islet somatostatin and PP is not well understood. PP was recently found to suppress glucagon secretion by direct effects on α-cells [2] and somatostatin inhibits both insulin and glucagon secretion [18]. Somatostatin pulsatility is in phase with that of insulin [20, 21], and somatostatin’s stronger inhibitory effect on glucagon than on insulin secretion [43] may help to shape pulsatile glucagon secretion in opposite phase [29].

Studies of cell functions and interactions in situ within pancreatic islets depend on unambiguous cell identification, which is far from trivial. Immunostaining of dispersed islet cells has successfully been used to identify all major islet cell types after studying their physiological Ca^2+^ responses [6, 19, 30, 31], but the process is time-consuming and cells may be lost during fixation and staining. The immunostaining approach is even more difficult when applied to cells within pancreatic islets, since it requires that the cells remain in exact position within the high magnification view field throughout the identification procedure. Electrophysiological techniques have also been used to identify α-, β-, and δ-cells [3, 8, 26] but this approach is difficult to apply to more than one cell at a time. The difference in expression of adrenergic receptors can also be utilized to discriminate α- and β-cells. α-cells mainly express α1- and β-receptors [44], which trigger elevations of the cytoplasmic Ca^2+^ and cAMP concentrations in response to adrenaline [49, 52]. In contrast, β-cells express mainly α2-receptors [44], and adrenaline therefore lowers cAMP [49] and suppresses Ca^2+^ signaling [52]. Similarly, the Ca^2+^ response to glutamate has been used for identifying α- and β-cells [9, 29] based on different expression of ionotropic glutamate receptors of AMPA/kainate type. These pharmacological tools depend on recordings of intracellular Ca^2+^ and cAMP. Moreover, they do not allow unambiguous separation of α- and β-cells from the less common δ- and PP-cells.

Cell identification by genetic labeling is an attractive alternative, where tissue-specific promoters are used to control expression of fluorescent markers. This approach has been applied to generate transgenic mice with selective expression of fluorescent proteins in α-, β-, and δ-cells under the promoters for preproglucagon, insulin, and somatostatin, respectively [1, 17, 38]. A drawback with this approach is that it is restricted to cell identification in specific transgenic mouse strains and not practically applicable to other species.

In the present study, we tested cell-type-specific promoter-driven expression of fluorescent proteins for identification of mouse and human islet cells in live-cell fluorescence microscopy applications. To ascertain sufficient expression levels, we constructed adenoviral vectors based on the Tet-On 3G conditional expression system [56, 32]. The preproglucagon promoter (Pppg), the rat insulin 2 promoter (Rip2), the somatostatin promoter (Psst), and the pancreatic polypeptide promoter (Pppy) were used to drive expression of the reverse tetracycline transactivator protein (rtTA), while the genes for the fluorescent proteins mCherry or enhanced green fluorescent protein (GFP) were placed downstream of the tetracycline response element. We demonstrate that this method results in mCherry or GFP expression in β-, α-, δ-, or PP-cells with a high degree of specificity and that appropriately labeled cells in mouse and human islets were amenable for on-line recordings of the sub-plasma membrane Ca^2+^ and cAMP concentrations ([Ca^2+^]_pm_ and [cAMP]_pm_).

## Materials and methods

### Isolation of cell-type-specific promoters

cDNA for Rip2 was a kind gift from Dr. Lena Stenson-Holst, Lund University. Pppg, Psst, and Pppy were isolated from genomic DNA using PCR. DNA was purified from MIN6 β-cells with PureLink® Genomic DNA Mini Kit (Thermo-Fisher Scientific, Waltham, MA, USA) and used as PCR template. Primer design was based on published promoter sequence data [11, 55, 22, 33, 25, 46, 47]. An XhoI restriction site (underlined) was added to the sense primer (5′-ATCGTACTCGAGGACACTCGCAATCATAAAGAGCAATC-3′ for Pppg; 5′-TATACTCGAGCAACCACTCCAAGTGGAG-3′ for Rip2; 5′-ATATATCTCGAGAGCCTAGAGGCAGAGCAAGCGCTG-3′ for Psst; 5′-CATGAGGCTCGAGTCGGAACTAGCCACTGGTTTTG-3′ for Pppy) and an AscI site to the antisense primer (5′-AAATGGCGCGCCTGAGCTGCGAACAGGTGTAG-3′ for Pppg; 5′-ATTTAAATGGCGCGCCTTACTGAATCCCCAC-3′ for Rip2; 5′-AATTAAGGCGCGCCGGAGACCGTGGAGAGCTCCATAGCG-3′ for Psst; 5′-GACATATGGCGCGCCTGTGCTGAGCTAGTGAGTG-3′ for Pppy). Standard PCR reactions were performed with PfuTurbo high-fidelity DNA polymerase (Agilent Technoliges, Santa Clara, CA, USA). The 1659-bp Pppg, 684-bp Rip2, 1986-bp Psst, and 1420-bp Pppy PCR products were purified by gel extraction, ligated into the pCR®II-TOPO vector (Thermo-Fisher Scientific, Waltham, MA, USA) and verified by sequencing (Eurofins Genomics, Ebergberg, Germany). Pppg, Rip2, Psst, and Pppy were subsequently released by digestion with XhoI and AscI.

### Construction of Tet-On 3G plasmids with cell-type-specific promoters

To avoid the potential problem that the promoters are too weak to drive expression of sufficient amounts of fluorescent protein for cell identification, we took advantage of the Tet-On conditional expression system. In this approach, a cell-type-specific promoter could drive expression of a small amount of rtTA, which is sufficient to strongly activate the tetracycline response element (TRE) and downstream genes in the presence of doxycycline.

To insert the cell-type-specific promoters in plasmids encoding rtTA, the pBR322-E3-Tet-On-rev plasmid [4] (kind gift from Prof Göran Akusjärvi, Uppsala University) was cleaved with XhoI and XbaI to release the cytomegalovirus promoter with a 5′UTR extension of the rtTA gene. This CMV-5′UTR fragment was subsequently ligated into an in-house made cloning vector denoted pCFH to get pCFH-CMV-5′UTR. After cleavage with XhoI and Eco53kI, the CMV promoter was released and replaced by the Rip2 promoter carrying a AscI site at the 3′ end. Eventually, the Rip2-5′UTR fragment was cut out with XhoI and XbaI and ligated into the cleaved pBR322-E3-rtTA-∆CMV fragment to get pBR322-E3-Rip2-rtTA, which now included the cell-type-specific promoter instead of the original CMV promoter. All other promoter constructs were completed by replacing Rip2 with Pppg, Psst, or Pppy, respectively, after XhoI-AscI double digestion. Finally, rtTA was replaced with Tet-On 3G transactivator [32, 56] by isolating the Tet-On 3G from pCMV-Tet3G plasmid (Clontech, USA) after digestion with XhaI and HpaI.

### Construction of plasmids for TRE-regulated mCherry and EGFP expression

In the next step, the pTRE3G-IRES plasmid (Clontech, Palo Alto, CA, USA) was cleaved with BglII and BamHI, to release the IRES component and insert either of the fluorescent proteins mCherry or GFP.

### Virus recombination and amplification

**Fig. 1:**
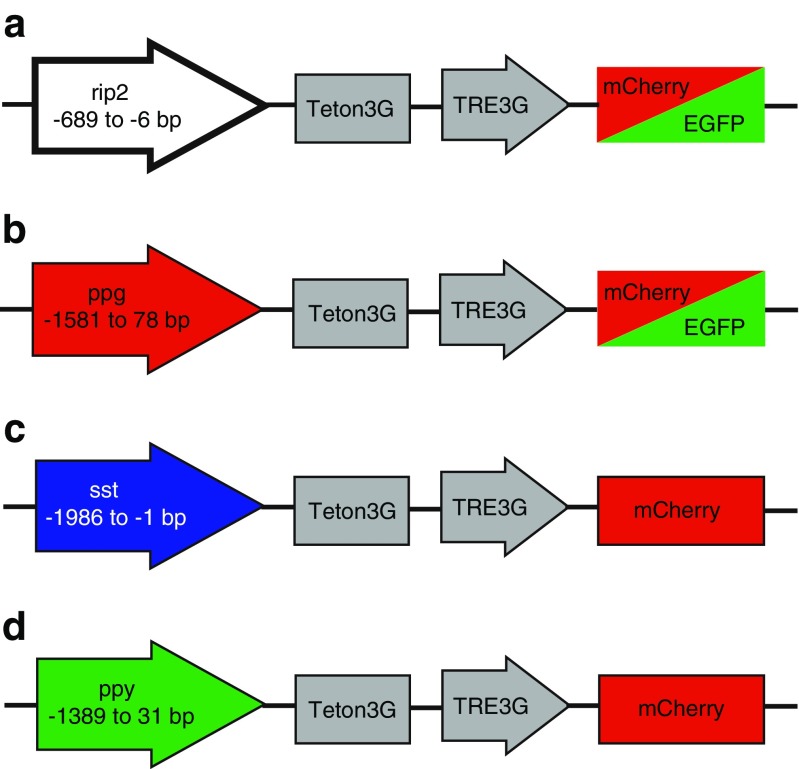
Adenoviral gene constructs for conditional, islet cell-type-specific expression of fluorescent proteins. The indicated fragments of the rat insulin 2 promoter (rip2), preproglucagon promoter (ppg), somatostatin promoter (sst), and the pancreatic polypeptide promoter (ppy) were inserted upstream of a Tet-On 3G element expressing the reverse tetracycline transactivator. In the presence of doxycycline, this gene product activates the tetracycline response element (TRE3G) and the associated promoter that drives expression of the fluorescent proteins mCherry or green fluorescent protein (GFP)

The four Tet-On 3G plasmids with cell-type-specific promoters and the two TRE plasmids with fluorescent protein were sent to Vector Biolabs (Philadelphia, PA, USA) for recombination and initial virus production. The final vector constructions are shown in Fig. 1. High titer virus stocks were created in-house in HEK 293 cells grown in 15 cm Petri dishes to about 50 % confluence. The cells were washed once with DMEM containing 2 % (*v*/*v*) fetal bovine serum (FBS) and infected with a titer of 2–5 MOI (multiplicity of infection) virus in this medium for 1 h. The infection mixture was subsequently removed and the cells were cultured in fresh DMEM containing 10 % (*v*/*v*) FBS for 2 or 3 days at 37 °C in a humidified atmosphere with 5 % CO_2_. When about 40 % of the cells had started to detach from the bottom of the Petri dish, all cells were harvested with a cell scraper, centrifuged for 7 min at 1800*g* and resuspended in 2 ml 0.1 M Tris-HCl, pH 8.0. Sodium deoxycholate (200 μl, 5 % *w*/*v*) was added and the cells were incubated on ice for 30 min. The virus was then released from the cells by sonication on ice three times for 10 s with 1-min intervals. The sonication solution was mixed with Tris-HCl and saturated CsCl and added to a 12.6-ml Quickseal tube (Beckman Instruments, Fullerton, CA, USA) and centrifuged for 16–20 h at 115,000*g*, 4 °C, with a Beckman 80Ti rotor. After centrifugation, the virus band was sucked out with a syringe and dialyzed for 4 h at 4 °C in phosphate buffered saline supplemented with 0.9 mM CaCl_2_ and 0.5 mM MgCl_2_ as well as 10 % (*v*/*v*) glycerol. After dialysis, the virus suspension was aliquoted and stored at −80 °C.

### Islet isolation, culture, and transduction

All procedures for animal handling and islet isolation were approved by the Uppsala animal ethics committee. Islets of Langerhans were isolated from C57B16J female mice as described previously [53]. After isolation, the islets were cultured for 1 to 2 days in RPMI-1640 medium containing 5.5 mM glucose, 10 % (*v*/*v*) fetal calf serum, 100 U/mL penicillin, and 100 μg/mL streptomycin at 37 °C in a 5 % CO_2_ humidified air atmosphere. Human islets were obtained from four normoglycemic cadaveric organ donors (two males, two females aged 57–65 years) via the Nordic Network for Clinical Islet Transplantation in Uppsala. All experiments with human islets were approved by the Uppsala human ethics committee. The isolated islets were cultured up to 7 days at 37 °C in an atmosphere of 5 % CO_2_ in CMRL 1066 culture medium containing 5.5 mM glucose, 100 U/mL penicillin and 100 μg/mL streptomycin, 2 mM glutamine, and 10 % FBS. For some experiments, the islets were dispersed into single cells and small clusters by transferring them to 2 mL cell dissociation buffer (Thermo Fisher Scientific) containing 10 % (*v*/*v*) trypsin-EDTA (0.05 %, Life Technologies), followed by pipetting up and down 20–30 times until all islets were disintegrated. The trypsin digestion was interrupted by addition of 8 ml serum-containing RPMI-1640 medium, followed by centrifugation for 5 min at 160*g* and resuspension of the cells in islet culture medium. The cell suspension was subsequently added onto poly-lysine-coated 25-mm coverslips and cultured overnight. The islets or cells were infected with adenovirus by 3 to 4 h exposure to a concentration of 105 fluorescence forming units (FFU)/islet [49], followed by addition of regular medium with 4 μM doxycycline and further culture for 16 to 20 h before use.

### Immunostaining

The infected islets were washed three times with PBS, fixed with 4 % (*w*/*v*) paraformaldehyde for 10 min in room temperature and permeabilized with 0.2 % (*v*/*v*) TrionX-100 for 10 min on ice. The reaction was blocked by adding PBS containing 5 % FBS in room temperature. After 30 min incubation, the primary antibody (polyclonal rabbit anti-insulin or polyclonal rabbit anti-glucagon from Invitrogen, Carlsbad, CA; polyclonal rabbit anti-somatostatin from Dako, Stockholm, Sweden; polyclonal goat anti-pancreatic polypeptide from Bio-Techne, Abingdon, UK) was added (1:200) for 2 h, followed by thorough rinsing with PBS. The secondary antibody, Alexa Fluor® 488 goat anti-rabbit IgG (Invitrogen, Carlsbad, CA) or Alexa Fluor® 488-AffiniPure F(ab’)2 fraction donkey anti-goat IgG (H + L) (Jackson ImmunoResearch Europe Ltd., Suffolk, UK), was then applied (1:200) for 1 h in darkness. After rinsing with PBS, the islets or cells were used for confocal microscopy imaging.

### Confocal microscopy

The islets or cells were imaged in a spinning-disk confocal system (Yokogawa CSU-10, Andor Technology, Belfast, Northern Ireland) attached to an Eclipse TE2000 microscope (Nikon, Kawasaki, Japan) equipped with a 60×, 1.40-NA objective (Nikon, Kawasaki, Japan). Diode-pumped solid-state lasers (Cobolt, Stockholm, Sweden) were used for excitation of mCherry (561 nm) and GFP or Alexa Fluor® 488 (491 nm). Fluorescence was selected with interference filters (520 with 35 nm half-bandwidth for GFP and Alexa Fluor® 488, and 586/20 nm for mCherry) and images were acquired with a back-illuminated EMCCD camera (DU888, Andor Technology) under MetaFluor software control (Molecular Devices Corp., Downington, PA). The confocal imaging experiments were performed at room temperature.

### Imaging of [cAMP]_pm_ and [Ca^2+^]_pm_

For imaging of [cAMP]_pm_, the islets were transduced with a cyan and yellow fluorescent protein (CFP and YFP)-based cAMP translocation biosensor [13] together with the fluorescent labeling vector and cultured over night. The islets or cells were then pre-incubated for 30 min at 37 °C in experimental buffer containing 125 mM NaCl, 4.8 mM KCl, 1.3 mM CaCl_2_, 1.2 mM MgCl_2_, and 25 mM HEPES (pH 7.40 set with NaOH) prior to imaging. For [Ca^2+^]_pm_ recordings, the islets were loaded with 1.3 μM of the Ca^2+^ indicator Fluo-4 during the pre-incubation period. After incubation, the islets were attached to poly-lysine-coated 25-mm coverslips and the coverslips with cells or islets were mounted in an open 50-μl chamber and superfused with experimental buffer at a rate of 0.3 ml/min. The chamber was attached to the stage of an objective-based total internal reflection fluorescence (TIRF) microscopy system consisting of an Eclipse Ti microscope (Nikon) with a 60×, 1.45-NA objective and a TIRF illuminator (Nikon) [49]. The chamber and superfusion medium was thermostated to 37 °C. The 457, 491, 515, and 561 nm lines of diode-pumped solid-state lasers (Cobolt) were used to excite CFP, Fluo-4, YFP, and mCherry, respectively. Fluorescence was detected with a back-illuminated EMCCD camera (DU-897, Andor Technology) controlled by MetaFluor. Emission wavelengths were selected with filters [485 nm/25 nm half-bandwidth for CFP, 527/27 nm for Fluo-4, 560/40 nm for YFP (Semrock Rochester, NY) and 620 nm long-pass for mCherry (Melles Griot, Didam, The Netherlands)] mounted in a filter wheel (Sutter Instruments). For time-lapse recordings, images or image pairs were acquired every 5 s with a shutter (Sutter Instruments) blocking the light between image captures. Image analysis was performed with MetaFluor. Ca^2+^ indicator fluorescence intensities under the plasma membrane were expressed as changes in relation to initial fluorescence intensity F_0_ after subtraction of background (F/F_0_). [cAMP]_pm_ was expressed as the ratio of CFP over YFP fluorescence after subtraction of background.

### Statistical analysis

Chi-square analysis with Yates correction was used to compare the proportion of cells that immunostained for a hormone in relation those with hormone promoter-controlled fluorescent protein expression.

## Results and discussion

### Fluorescence labeling of β-cells in mouse and human pancreatic islets

**Fig. 2:**
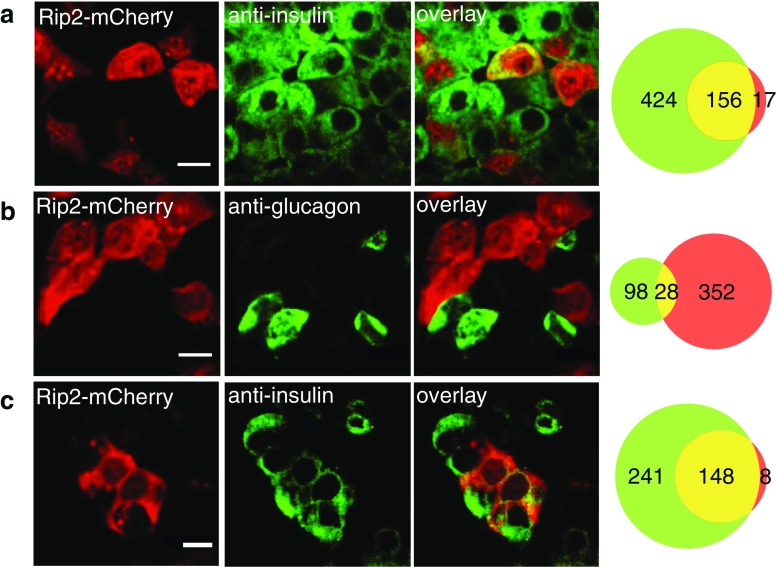
Insulin promoter-controlled expression of mCherry in mouse and human islet cells. Confocal microscopy images of Rip2-controlled mCherry expression and insulin or glucagon immunostaining in mouse and human islets. **a** Rip2-mCherry expressing and insulin positive cells in a mouse islet. **b** Rip2-mCherry expressing and glucagon positive cells in a mouse islet. **c** Rip2-mCherry expressing and insulin positive cells in a human islet. *Scale bars*, 10 μm. The Venn diagrams on the right show the total number of cells with hormone staining (*green*), mCherry expression (*red*), and both (*yellow*)

Isolated mouse islets were transduced with adenovirus expressing GFP or the red fluorescent protein mCherry conditionally controlled by the Rip2 promoter and the Tet-On 3G system and subsequently imaged with confocal microscopy. Fluorescence was absent in islets that had not been exposed to doxycycline (not shown), indicating that there is minimal leak expression with this conditional vector system. Islets treated overnight with 4 μM doxycycline showed bright GFP (not shown) or mCherry fluorescence (Fig. 2a). The specificity of the targeting strategy was evaluated using the mCherry-expressing vectors. Transduced islets immunostained for insulin showed cells with red (mCherry) and green (insulin) fluorescence (Fig. 2a). As indicated by the Venn plot, 97 % of the fluorescent cells stained for insulin, 28 % were mCherry positive and 26 % displayed both labels. When islets with induced Rip2-mCherry expression were instead immunostained for glucagon only 6 % of the fluorescent cells showed both labels (Fig. 2b). mCherry expression is apparently a good predictor of β-cell identity since 90 % of the red cells stained for insulin and only 7 % for glucagon. When Rip2-mCherry expression was induced in dispersed islet cells that were then immunostained for insulin, 32 fluorescent cells all stained for insulin and 78 % (25 cells) were also mCherry positive (not shown). The much higher proportion of mCherry label in dispersed fluorescent cells as compared to those within islets (78 vs 28 %; *P* < 0.001) is probably due to restricted virus access to the islet interior where most β-cells are located [45]. The reason for the expression of Rip2-mCherry in a small number of glucagon-positive islet cells and why no such mistargeting was found in dispersed cells is unclear. Human β-cells in intact islets could be identified by the same approach, since 95 % of mCherry expressing cells also stained for insulin (Fig. 2c), and the corresponding fraction was 93 % among dispersed human islet cells (28 of 30 cells, not shown).

### Fluorescence labeling of α-cells in mouse and human pancreatic islets

**Fig. 3:**
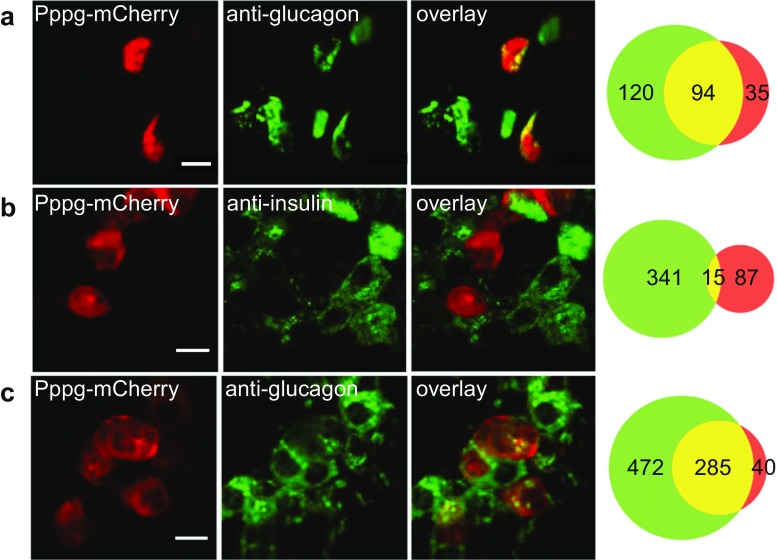
Glucagon promoter-controlled expression of mCherry in mouse and human islet cells. Confocal microscopy images of Pppg-controlled mCherry expression and insulin or glucagon immunostaining in mouse and human islet cells. **a** Pppg-mCherry expressing and glucagon positive cells in a mouse islet. **b** Pppg-mCherry expressing and insulin positive cells in a mouse islet. **c** Pppg-mCherry expressing and glucagon positive cells in a human islet. *Scale bars*, 10 μm. The Venn diagrams on the right show the total number of cells with hormone staining (*green*), mCherry expression (*red*), and both (*yellow*)

Mouse islets transduced with adenovirus containing Pppg and mCherry or GFP showed bright red or green fluorescent cells. As with Rip2, evaluation of targeting specificity was made with the mCherry-expressing virus. In transduced mouse islets immunostained for glucagon, 52 % of the fluorescent cells were mCherry positive (Fig. 3a). This proportion is higher than for the β-cells (28 %, *P* < 0.001), probably because α-cells are more easily transduced with virus in being located in the periphery of rodent islets [45]. mCherry expression was a good predictor of α-cell phenotype since 73 % of the cells within rodent islets also stained for glucagon, and the corresponding fraction was 100 % among dispersed islet cells (all of 64 cells, not shown). After instead staining for insulin 15 % of the mCherry expressing cells within islets were positive (Fig. 3b). This mismatch was higher than for β-cells (7 %, *P* < 0.05) and it is unclear whether it reflects incomplete specificity of the relatively short Pppg sequence and/or a small population of cells with ambiguous identity. It has been increasingly recognized that islet cells show plasticity with transdifferentiation between α- and β-cells under certain conditions [48, 51, 7]. The Pppg adenovirus readily transduced human islets and following glucagon immunostaining, 41 % of all fluorescent cells were mCherry positive. This proportion tended to be lower than in mouse islets (52 %, *P* = 0.068), which is expected since human α-cells are not preferentially located in the islet periphery [45]. Among the mCherry-expressing cells within islets, 88 % were verified as α-cells by glucagon immunostaining (Fig. 3c), and the corresponding fraction was 85 % for dispersed human islet cells (47 of 55 cells, not shown).

### Fluorescence labeling of mouse and human δ- and PP-cells

**Fig. 4:**
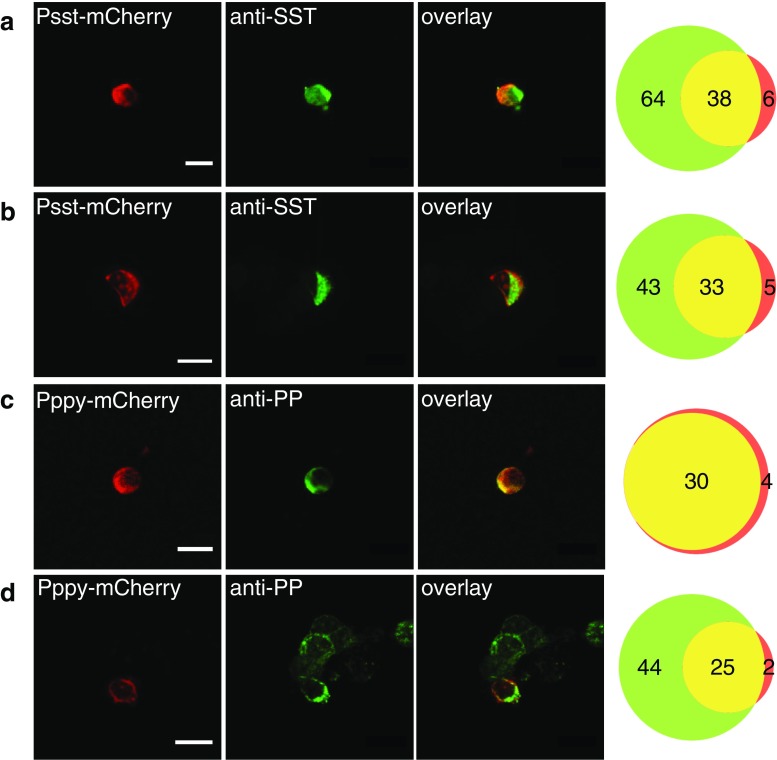
Somatostatin and PP promoter-controlled expression of mCherry in mouse and human islet cells. Confocal microscopy images of Psst- and Pppy-controlled mCherry expression and somatostatin and PP immunostaining in dispersed mouse and human islet cells. **a**, **b** Psst-mCherry expressing and somatostatin positive mouse (**a**) and human (**b**) islet cells. **c**, **d** Pppy-mCherry expressing and PP positive mouse (**c**) and human (**d**) islet cells. *Scale bars*, 10 μm. The Venn diagrams on the right show the total number of cells with hormone staining (*green*), mCherry expression (*red*), and both (*yellow*)

The adenovirus with Psst and Pppy transduced relatively few cells in mouse and human islets. To effectively find these cells, we transduced and immunostained dispersed mouse and human islet cells. The Psst-mCherry expression overlapped with positive anti-somatostatin staining in 86–87 % in both species, verifying their identity as δ-cells (Fig. 4a, b). Labeling with Pppy-mCherry was observed both in mouse (Fig. 4c) and human (Fig. 4d) PP-cells, whose identity was verified by positive PP-immunostaining in 88 % of the mouse and 93 % of the human cells.

### TIRF recordings of [Ca^2+^]_pm_ in islet cells identified by cell-type-specific fluorescent protein expression

We next tested whether the cell identification strategy could be used in live-cell imaging applications. Islets transduced with the respective cell-type-specific vector expressing mCherry were loaded with the green fluorescent Ca^2+^ indicator fluo-4, which does not show spectral overlap with the red label. The islets were imaged with a total internal reflection fluorescence microscope, which excites a small volume within <100 nm of the plasma membrane of the cells in contact with the coverslip. Although this technique does not enable analysis of cells located deeper in the islet, it is advantageous in allowing recordings from small cells with weak fluorescence without contamination from cells located in a different focal plane.

**Fig. 5:**
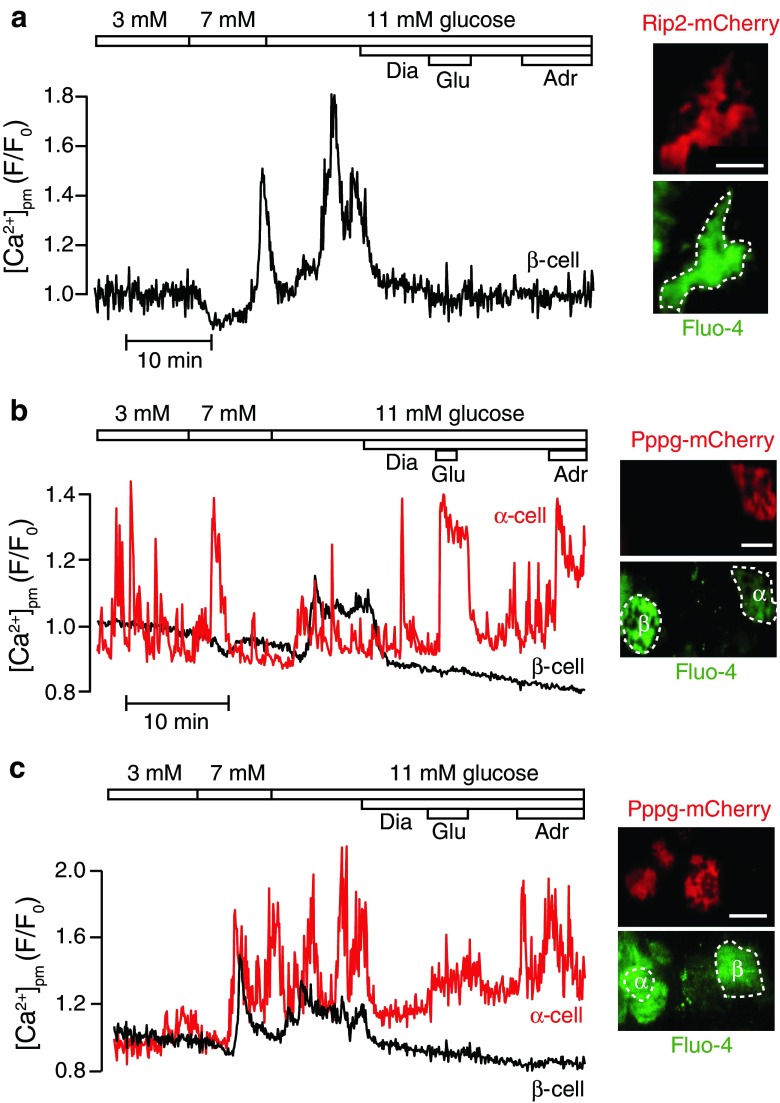
Recordings of sub-plasma membrane Ca^2+^ concentration in β- and α-cells identified by cell-type-specific promoter-controlled expression of mCherry. TIRF microscopy recordings of [Ca^2+^]_pm_ in cells within intact mouse islets loaded with the Ca^2+^ indicator Fluo-4 and exposed to 3–11 mM glucose, 250 μM diazoxide (Diaz), 1 mM glutamate (Glu), and 10 μM adrenaline (Adr). **a** [Ca^2+^]_pm_ recording from a β-cell identified by Rip2-mCherry expression. Increase of the glucose concentration from 3 to 7 mM induces elevation of [Ca^2+^]_pm_, which is reversed by diazoxide. Glutamate and adrenaline are without effect on basal [Ca^2+^]_pm_. Representative of 18 cells from five islets. **b** [Ca^2+^]_pm_ recording from an α-cell identified by Pppg-mCherry expression and an adjacent β-cell identified by the cell-type-specific [Ca^2+^]_pm_ response pattern. Irrespective of the glucose concentration, the α-cell shows irregular [Ca^2+^]_pm_ oscillations which are not interrupted by diazoxide. Glutamate and adrenaline increases [Ca^2+^]_pm_ in the α-cell. The β-cell shows typical [Ca^2+^]_pm_ elevation in response to 11 mM glucose. Representative of 7 out of 10 α-cells in five islets. **c** [Ca^2+^]_pm_ recording from another α-cell identified by Pppg-mCherry expression and an adjacent β-cell identified on the typical response pattern. The α-cell shows β-cell-like responses with glucose-induced elevation of [Ca^2+^]_pm_ that was reversed by diazoxide. However, in contrast to the β-cell, the α-cell readily responds to glutamate and adrenaline. Representative for 3 out of 10 α-cells in five islets. *Scale bars*, 10 μm

We stimulated the islets with various concentrations of glucose and tested the responses to the K_ATP_ channel opener diazoxide and the neurotransmitters glutamate and adrenaline, which have been used to discriminate between the different islet cell types [9, 29, 49, 52]. Mouse β-cells identified by their Rip2-controlled mCherry fluorescence showed low and stable [Ca^2+^]_pm_ in the presence of 3 mM glucose (Fig. 5a). Increase of the glucose concentration to 7 mM resulted in lowering of [Ca^2+^]_pm_, known to reflect stimulated uptake of Ca^2+^ into the endoplasmic reticulum, followed after a few minutes delay by an increase reflecting voltage-dependent Ca^2+^ influx [10]. Further elevation of glucose to 11 mM resulted again in transient lowering followed by rise of [Ca^2+^]_pm_ with typical slow oscillations that underlie pulsatile insulin secretion. Application of the hyperpolarizing agent diazoxide (250 μM) interrupted the voltage-dependent Ca^2+^ influx and [Ca^2+^]_pm_ levels returned to baseline. Under these conditions, the β-cells did not respond to 1 mM glutamate or 10 μM adrenaline (Fig. 5a). The observed response pattern is characteristic of β-cells and there was not much variability between different cells. From these observations, we conclude that the viral vector and fluorescent protein expression do not interfere with β-cell function.

### Variability of [Ca^2+^]_pm_ responses to glucose in α-cells

Recordings from α-cells identified by Pppg-mCherry fluorescence were largely consistent with a previous study of α-cell [Ca^2+^]_pm_ signaling [29]. Accordingly, most cells showed irregular brief [Ca^2+^]_pm_ oscillations in the presence of 3 mM glucose, and increases of the glucose concentration to 7 and 11 mM did not induce sustained suppression of [Ca^2+^]_pm_ signaling (Fig. 5b) but there was sometimes a transient interruption of [Ca^2+^]_pm_ oscillations or somewhat lower amplitude of the oscillations at 11 mM compared to 3 mM glucose. Seven out of 10 α-cells showed no or weak response to K_ATP_ channel activation with diazoxide (Fig. 5b), supporting the view that this channel may not be required for glucose regulation of glucagon secretion [14]. In accordance with previous observations [29], the mouse α-cells responded to 1 mM glutamate with a distinct, stable [Ca^2+^]_pm_ elevation (Fig. 5b). Also, 10 μM adrenaline induced [Ca^2+^]_pm_ elevation but this response was usually weaker and/or more transient than that to glutamate. mCherry-negative cells from the same islets showed β-cell-like responses with low and stable [Ca^2+^]_pm_ at 3 mM glucose, a distinct increase of [Ca^2+^]_pm_ at 11 mM, which was reversed by diazoxide, and no responses to glutamate or adrenaline (Fig. 5b). Our genetic labeling strategy thus mostly identifies α- and β-cells with characteristic [Ca^2+^]_pm_ responses to low and high glucose concentrations as well as to glutamate and adrenaline.

A few mCherry-positive α-cells showed somewhat more β-cell-like response with absence of prominent [Ca^2+^]_pm_ signaling activity at 3 mM glucose and distinct [Ca^2+^]_pm_ increases after elevation of glucose to 7 mM (Fig. 5c). The two types of α-cell responses were sometimes observed in different cells within the same islet. In the cell shown in Fig. 5c, the glucose-induced [Ca^2+^]_pm_ elevation was reversed by diazoxide, like in β-cells. One may speculate that this type of response represents “mislabeled” cells with Pppg-controlled mCherry expression and positive immunostaining for insulin. However, the cell shown in Fig. 5c responded to glutamate and adrenaline like a typical α-cell. Simultaneous recording from an adjacent mCherry negative cell exhibited the typical β-cell behavior with glucose-induced [Ca^2+^]_pm_ increases and lack of glutamate and adrenaline responses. The results indicate possible presence of sub-populations of α-cells that respond differently to glucose. Although glucose is generally an inhibitor of glucagon release, secretion from purified rodent α-cells is paradoxically stimulated by the sugar [23, 28, 35]. One possibility is that purification by FACS sorting somehow selects for the α-cells with β-cell-like Ca^2+^ responses and glucose-stimulated glucagon secretion, although the α-cells with glucose-inhibited secretion dominate within the islet. However, there is also evidence that Ca^2+^ signaling may differ between isolated α-cells and those within islets [29, 53] and that glucagon release is controlled by factors that may override a stimulatory [Ca^2+^]_pm_ signal [28, 29]. It is pertinent to note that the dose-response relationship for glucose-regulated glucagon secretion is U-shaped with maximal inhibition at 7 mM glucose. At higher concentrations of the sugar, inhibition is gradually diminished and secretion even stimulated [28, 41]. It remains to establish if the population of α-cells with [Ca^2+^]_pm_ increase in response to glucose elevation may perhaps explain the stimulatory glucose component. We conclude that our labeling approach dominatingly selects for α-cells with expected [Ca^2+^]_pm_ responses to glutamate and adrenaline and reveals a considerable variability in the α-cell reactivity to glucose.

### [Ca^2+^]_pm_ signaling in mouse δ-cells

**Fig. 6:**
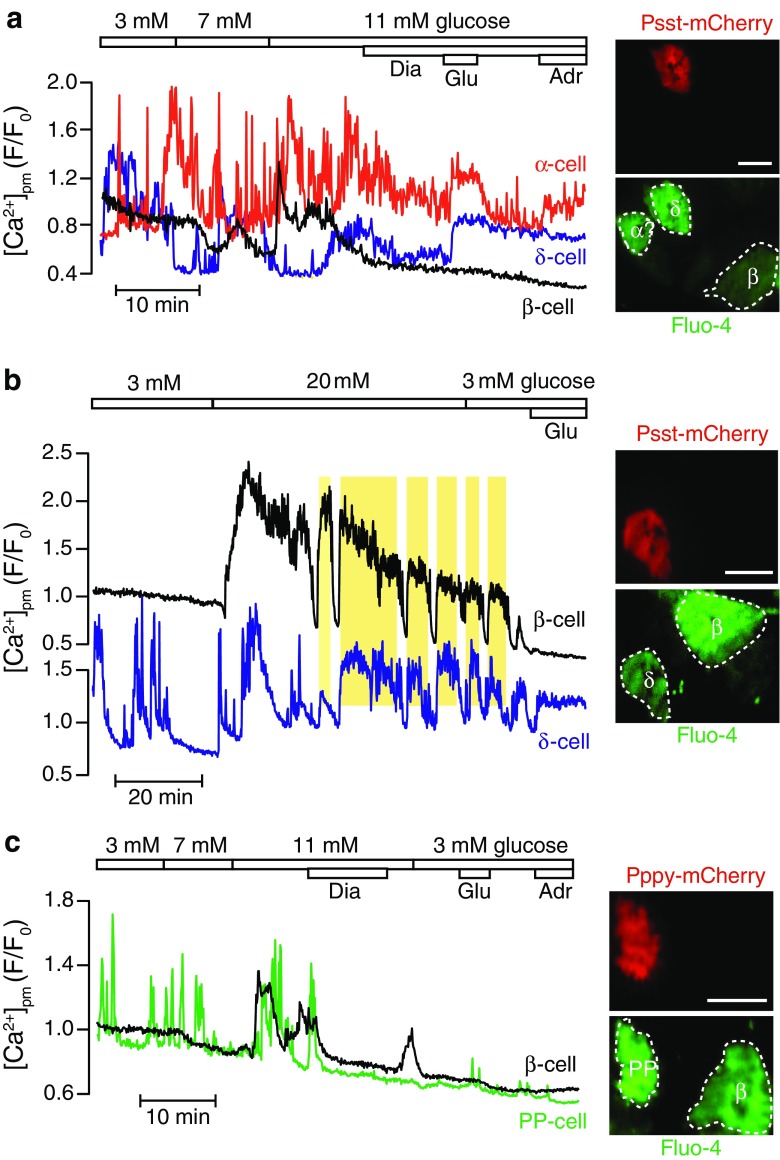
Recordings of [Ca^2+^]_pm_ in islet cells identified by cell-type-specific promoter-controlled expression of mCherry. TIRF microscopy recordings of [Ca^2+^]_pm_ in cells within intact mouse islets loaded with Fluo-4. **a** [Ca^2+^]_pm_ recording from a δ-cell identified by Psst-mCherry expression and adjacent α- and β-cells identified by typical response patterns. The δ-cell shows irregular [Ca^2+^]_pm_ elevations already at low glucose concentrations and diazoxide lowers [Ca^2+^]_pm_, while glutamate induces an increase. Representative of 8 δ-cells from six islets. **b** [Ca^2+^]_pm_ recording from a Psst-mCherry-identified δ-cell and an adjacent β-cell with typical response pattern. In the presence of high glucose, the β-cell shows regular [Ca^2+^]_pm_ oscillations that are synchronized with those in the δ-cell. **c** [Ca^2+^]_pm_ recording in a PP-cell identified by Pppy-mCherry expression showing that the cell is active already at low glucose concentrations. Representative of three PP-cells from three islets. *Scale bars*, 10 μm

[Ca^2+^]_pm_ recordings in islet cells identified as δ-cells by Psst-controlled mCherry expression showed fast and irregular [Ca^2+^]_pm_ oscillations in the presence of 3 mM glucose, thus reminiscent of the responses in α-cells (Fig. 6a). The pattern was not clearly changed by elevating glucose to 7 or 11 mM. Diazoxide reduced [Ca^2+^]_pm_, consistent with the involvement of K_ATP_ channels in δ-cell electrical activity [5, 8, 16]. Glutamate induced prompt [Ca^2+^]_pm_ elevation in δ-cells but there was no clear effect of adrenaline. In addition to the genetically labeled δ-cell, Fig. 6a shows [Ca^2+^]_pm_ recordings from two unlabeled cells, which based on the [Ca^2+^]_pm_ signaling patterns probably are α- and β-cells.

Another example of δ-cell [Ca^2+^]_pm_ signaling is shown in Fig. 6b. Apart from the mCherry-expressing δ-cell, the recording includes a non-expressing β-cell. When the glucose concentration was increased from 3 to 20 mM, the β-cell shows the typical transition from silence at low glucose to [Ca^2+^]_pm_ elevation followed by quite regular, slow oscillations and [Ca^2+^]_pm_ reaches baseline within 10 min after restoration of glucose to 3 mM. In contrast, the δ-cell is active at 3 mM glucose with no major change after introduction of 20 mM glucose. After approximately 10 min, the [Ca^2+^]_pm_ oscillations in the δ-cell become well synchronized with those in the β-cell (Fig. 6b). The temporal relationship between [Ca^2+^]_pm_ oscillations in β- and δ-cells within intact islets have previously not been clarified, but since pulsatile release of insulin and somatostatin is synchronized in phase [21], the prediction is that [Ca^2+^]_pm_ oscillations are synchronized in the same phase. Similar synchronization was recently found also between β- and α-cells, although insulin and glucagon pulses are synchronized in opposite phase [29]. This apparent paradox may be explained by release of somatostatin that suppresses glucagon secretion with little influence on α-cell [Ca^2+^]_pm_.

### [Ca^2+^]_pm_ signaling in identified PP-cells

PP-cells identified by Pppy-mediated expression of mCherry were also amenable for [Ca^2+^]_pm_ recordings. Figure 6c shows an example of a PP-cell exhibiting [Ca^2+^]_pm_ oscillations in the presence of 3 mM glucose, which did not change appearance when glucose is increased to 7 or 11 mM. However, diazoxide promptly interrupted the signaling. There is only a weak response to glutamate and no consistent response to adrenaline (Fig. 6c).

### Imaging cells co-expressing genetically encoded biosensors and an identity marker

**Fig. 7:**
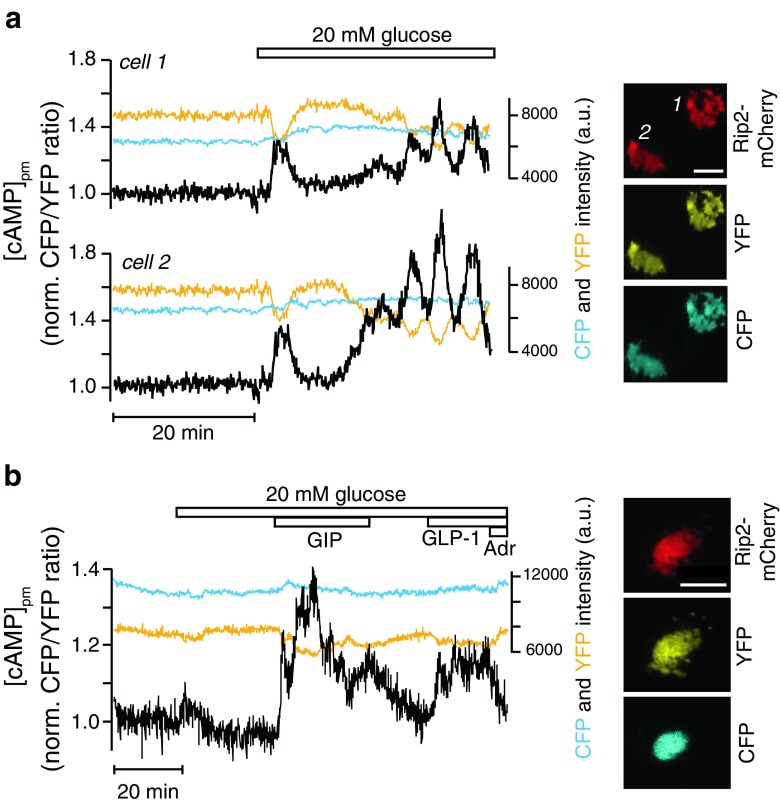
Recordings of [cAMP]_pm_ in islet β-cells identified by cell-type-specific promoter-controlled expression of mCherry. TIRF microscopy recordings of [cAMP]_pm_ in β-cells within intact islets identified by Rip2-mCherry expression. The images show expression of Rip2-mCherry and the CFP- and YFP-labeled components of the cAMP reporter. [cAMP]_pm_ is expressed as the CFP/YFP intensity ratio with the prestimulatory level normalized to unity (*black traces*). The *blue* and *yellow traces* show the CFP and YFP fluorescence intensity raw data. **a** [cAMP]_pm_ recordings from two mouse β-cells in the same islet. Elevation of the glucose concentration from 3 to 20 mM induces synchronized, oscillatory increases of [cAMP]_pm_. Representative of 40 cells from 10 islets. **b** [cAMP]_pm_ recording from a human β-cell during elevation of the glucose concentration from 3 to 20 mM followed by addition of 100 nM GIP, 100 nM GLP-1, and 10 μM adrenaline (Adr). Representative of four cells from three islets from one donor. *Scale bars*, 10 μm

We next tested whether the fluorescent protein labeling strategy could be used also in cells simultaneously expressing a genetically encoded biosensor. We transduced mouse or human islets with adenovirus for Rip2-mCherry and a CFP/YFP-based biosensor for [cAMP]_pm_ [12]. Figure 7a shows a [cAMP]_pm_ recording in two β-cells during an increase of the glucose concentration from 3 to 20 mM. Both cells show an initial [cAMP]_pm_ elevation followed by a decline and a more pronounced increased with slow oscillations that are synchronized between the two cells. Since the two cells apparently lack direct physical contact, the synchronization may be explained by indirect coupling due to extensive gap junctions between most β-cells within the islet [39]. We also transduced human islets with the Rip2-mCherry virus. The β-cell in Fig. 7b shows a very modest [cAMP]_pm_ elevation in response to 20 mM glucose but pronounced [cAMP]_pm_ increases after stimulation with 100 nM of the gluco-incretin hormones GIP (glucose-dependent insulinotropic polypeptide) and GLP-1 (glucagon-like peptide 1). The recording also demonstrates the β-cell typical reduction of [cAMP]_pm_ after exposure to 10 μM adrenaline (Fig. 7b).

## Concluding remarks

We have created a set of viral vectors that allow pancreatic islet cell-specific expression of fluorescent proteins for their identification in various live-cell imaging applications. These vectors provide a valuable addition to the toolbox for live-cell imaging applications. In such settings, fluorescent cells are easily identified and simultaneous recordings can often be made on several cells. We first applied the tools with [Ca^2+^]_pm_ recordings to verify that cells responded normally to well-known stimuli and previously described functional identification criteria. These initial measurements highlighted a previously overlooked [Ca^2+^]_pm_ response pattern in certain α-cells and indicated that [Ca^2+^]_pm_ oscillations in β-cells are synchronized with those in δ-cells. We also demonstrated that the labeling strategy can be combined with genetically encoded fluorescence biosensors as exemplified by intracellular cAMP recordings. This approach is considerably more flexible than cell-type-specific expression of biosensors. The potential of this system could be further improved using an infrared fluorescent protein for identification, and leave the visual spectral channels for functional biosensors.
